# Biomarker signature identification in “omics” data with multi-class outcome

**DOI:** 10.5936/csbj.201303004

**Published:** 2013-06-08

**Authors:** Vincenzo Lagani, George Kortas, Ioannis Tsamardinos

**Affiliations:** a Institute of Computer Science, Foundation for Research and Technology – Hellas (FORTH), N. Plastira 100, Vassilika Vouton, GR-700 13 Heraklion, Crete, Greece; b Department of Computer Science, University of Crete, P.O.Box 2208, GR-710 03 Heraklion, Crete, Greece

**Keywords:** Constraint-based Methods, Graphical Models, Biomarker Signature Identification, Multiple Outcomes Studies, High Dimensional Data, “Omics” Data

## Abstract

Biomarker signature identification in “omics” data is a complex challenge that requires specialized feature selection algorithms. The objective of these algorithms is to select the smallest set(s) of molecular quantities that are able to predict a given outcome (target) with maximal predictive performance. This task is even more challenging when the outcome comprises of multiple classes; for example, one may be interested in identifying the genes whose expressions allow discrimination among different types of cancer (nominal outcome) or among different stages of the same cancer, e.g. Stage 1, 2, 3 and 4 of Lung Adenocarcinoma (ordinal outcome). In this work, we consider a particular type of successful feature selection methods, named constraint-based, local causal discovery algorithms. These algorithms depend on performing a series of conditional independence tests. We extend these algorithms for the analysis of problems with continuous predictors and multi-class outcomes, by developing and equipping them with an appropriate conditional independence test procedure for both nominal and ordinal multi-class targets. The test is based on *multinomial logistic* regression and employs the log-likelihood ratio test for model selection. We present a comparative, experimental evaluation on seven real-world, high-dimensional, gene-expression datasets. Within the scope of our analysis the results indicate that the new conditional independence test allows the identification of smaller and better performing signatures for multi-class outcome datasets, with respect to the current alternatives for performing the independence tests.

## Introduction

Microarray technologies and Next Generation Sequencing (NGS) techniques nowadays allow precise measurements of several types of within-cell molecular quantities. Gene expression data, methylation level measurements, proteomics and metabolomics information are just a few example of the “omics” data that such technologies are able to provide.

Even though “omics” technologies simultaneously measure tens of thousands or more molecular quantities, researchers are often interested in identifying a relatively small subset of such measurements, known as *biomarkers*, which are relevant for the problem under study. A typical example is the identification of genes whose expressions statistically significantly differ among two or more conditions (i.e., differentially expressed genes).

Identifying biomarkers that are relevant (informative) when considered *in isolation* is often useful for investigating biological systems’ underlying mechanisms. However, in some cases the identification of *biomarker signatures* is necessary instead. We define a biomarker signature as a minimal subset of molecular quantities that are maximally informative for a given task when considered jointly.

For example, a researcher may be interested in finding the smallest subset of genetic variants (e.g., Single Nucleotide Polymorphisms, SNPs) that considered together are maximally predictive with respect to the development of osteoporosis in elderly women. As a further example, one may focus on finding the smallest number of CpG sites whose methylation levels discriminate with the maximal possible accuracy among different types of lung cancers. In both examples it is crucial to identify the set of biomarkers providing the highest possible accuracy; at the same time, it is necessary to take into account that the cost of devising, realizing and routinely performing a clinical test based on the signature is probably directly related to the number of involved biomarkers.

A widely adopted approach for identifying new biomarker signatures consists in measuring a large set of molecular quantities from a sufficiently large sample of biological specimens, and then employing data-analysis approaches in order to select the most informative set of features.

In the fields of statistics and machine learning the task of identifying the most relevant variables for the problem at hand is known as *feature* or *variable selection* [1]. Numerous methods have been developed for addressing the problem. A recent successful approach based on local causal discovery, namely the Max-Min Parent Children (MMPC) algorithm, has been described in [2]. Unlike some other methods, this approach is principled in the sense that it provides theoretical guarantees under which the methods soundly solve the feature selection problem. In particular, MMPC attempts to retrieve (a subset of) the *Markov-Blanket* (MB) of the considered outcome. The MB of a target variable is the set of variables conditioned upon which any other set of variables becomes independent by the target. It has been theoretically demonstrated that, under broad assumptions, the MB of a variable coincides with its minimal-size, most-informative signature [3]. This theoretical result was recently supported by large scale evaluations [4, 5] that have experimentally demonstrated local causal discovery algorithms’ efficacy in finding highly predictive signatures.

MMPC operates as a Constraint-Based (CB) variable selection algorithm. The operation of all such CB methods iteratively applies, based on a search strategy, *Conditional Independence Tests* (CITs) for characterizing the data distribution and identifying the variables (not) belonging to the MB. CITs, hereafter represented as Test(X, Y ∣**Z**), are statistical procedures that assess the null hypothesis “X and Y are independent given **Z**”, where X and Y are two random variables, the conditioning set **Z** is a (possibly empty) set of random variables, and X,Y ∉ **Z**. Intuitively, a CIT assesses whether X gives any additional information for Y (and vice-versa) once **Z** is known.

CITs role within CB algorithms is pivotal; employing an inappropriate CIT would lead to a poorly approximated MB and consequently to a suboptimal signature. The Fisher Z test [6] is currently the most widely employed conditional independence test for cases when all variables, including the target, are continuous. The Fisher test assumes linear relations among variables as well as normally distributed error terms: assumptions that are quite unlikely to hold in omics data. For discrete data testing is typically implemented with asymptotic G^2^ and χ^2^ tests [7] or exact permutation-based versions of these tests [8]. Attempts have been performed in order to develop sample-efficient CIT not relying on parametric assumptions [9], but further research in this field is needed: in particular, large scale evaluations for comparing different CITs’ respective performances are largely missing. To the best of our knowledge, up to date no CIT for continuous predictors / multi-class target has been applied and evaluated for CB algorithms.

In this paper, we devise a CIT specifically for cases where all predictors are numerical (continuous) and the target outcome represents multiple classes (categories). This is a common scenario in studies dealing with “omics” data that look for molecular signatures able to discriminate among different conditions (e.g., different cancer stages or types). The Fisher Z test is usually employed in these settings, after encoding the outcome as a discrete, integer variable; this workaround introduces a possibly unnatural order among outcome categories and assumes linear relationships among regressors and outcome. The CIT we develop, named Multi-Class Conditional Independence Test (MC-CIT) is based on the multinomial logistic regression and is turned into a test by employing the log-likelihood ratio test for model selection [10]. The multinomial Logistic models are Generalized Linear Models (GLM [11]) specifically devised for modeling multi-class outcomes; we thus expect MC-CIT to outperform the Fisher Z test in such settings.

In order to support our claim, we contrasted the newly proposed test against both the prototypical Fisher Z and G^2^ tests, in an extensive evaluation involving seven high-dimensional, multi-class gene expression studies. The following sections describe MC-CIT theoretical basis and implementation details, along with the experimentation protocol employed for assessing its performances.

Notably, the results of the experimentation underlined the superior performances of MC-CIT against the Fisher Z test, in terms of both predictive capability and parsimoniousness of the selected biomarker signatures.

## Experimental procedures

### MC-CIT: Conditional Independence Test based on Multinomial Logistic Regression

Let's assume that Y is a categorical random variable representing a multi-class outcome, X a continuous random variable and **Z** a set of continuous random variables; let's indicate with *Ind*(X,Y∣**Z**) the MC-CIT null hypothesis of independence “Y is independent by X given **Z**”. Assuming that *Ind*(X,Y∣**Z**) holds implies that X is not necessary for predicting Y once **Z** is known; under this respect the MC-CIT can be devised as a nested-model selection procedure, where the “full” model employs {X, **Z**} as regressors for Y, while the alternative model employs only {**Z**} [12]. When the full model shows a statistically significantly better fit than the alternative model, then the null hypothesis *Ind*(X,Y∣**Z**) can be rejected, i.e., X and Y are *associated* given **Z**. When the two models are statistically indistinguishable then the null hypothesis cannot be rejected and the algorithm accepts that the conditional independence holds.

Following these considerations, we implemented the MC-CIT as a log-likelihood ratio test for nested-model selection, where the full and alternative models are fitted with either a Multinomial Logistic (ML, for categorical outcomes) or with an Ordered Logit (OL [11], for ordered outcomes) regression approach. In both cases, let *LogL*_*Full*_ and *LogL*_*Altern*_ be the log-likelihood of the full and alternative model, respectively; then the quantity *D*

$$D=-2\cdot \left(\mathrm{Log}L_{\mathrm{Full}}-\mathrm{Log}L_{\mathrm{Altern}}\right)$$

follows a χ^2^ distribution with one degree of freedom, under the assumption that *Ind*(X,Y∣**Z**) holds. Given D and its theoretical distribution we calculate a p-value for the MC-CIT null hypothesis.

The key reason for preferring the ML and OL regressions over the simpler Fisher Z test linear approach is that these two regression procedures are specifically devised in order to model discrete outcomes over continuous regressors. We thus expect ML and OL to better model multi-outcome data and consequently to enhance the detection of true conditional dependencies. Specifically, given a set of N regressors **W** and a binary outcome T, the standard logistic regression model can be expressed as:

$$\ln\left(\frac{\mathrm{pr}\left(T_{i}=1\right)}{1-\mathrm{pr}\left(T_{i}=1\right)}\right)=\beta \cdot W_{i}$$

where **β** is the set of model's coefficients, **W**_i_ represents the values that the regressors assume for the i^th^ sample, and Pr(T_i_ = 1) is the probability that T_i_ = 1.

The ML regression extends the standard logistic regression to categorical outcomes by introducing K-1 set of coefficient **β**_k_, where K is the number of different values that the outcome can assume:

$$\text{ln}\left(\frac{\text{Pr}\left(T_{i}=k\right)}{\text{Pr}\left(T_{i}=K\right)}\right)=\beta_{k}\cdot W_{i},\;k=1,\cdots ,K-1$$

Few simple mathematical operations bring to the following formulation:

$$\text{Pr}\left(T_{i}=k\right)=\text{Pr}\left(T_{i}=K\right)\cdot e^{\beta_{k}\cdot W_{i}},\;k=1,\cdots ,K-1$$

The probability Pr(T_i_ = K) can be calculated by taking in consideration that the whole set of probabilities Pr(T_i_ = k), k = 1, …, K must sum up to 1:

$$\text{Pr}\left(T_{i}=K\right)=\frac{1}{1-\sum_{k=1}^{K-1}e^{\beta_{k}\cdot W_{i}}}$$

In short, for a given sample the ML regression employs K – 1 different equations for estimating the probability of belonging to each outcome class.

For ordinal outcomes we adopted a different extension of the logistic regression, the Ordered Logit models. The principle behind OL models is that the outcome T is supposed to be the observable realization of a latent variable T*. The relationships between T and T* is governed by the following set of equations:

$$T=\{\begin{array}{c} 1\;\mathrm{if}\;T^{*}\leq \mu_{1}\\ 2\;\mathrm{if}\;\mu_{1}<T^{*}\leq \mu_{2}\\ \cdots \\ K\;\mathrm{if}\;T^{*}>\mu_{k-1} \end{array}$$

where **µ** is a set of K -1 coefficients to be estimated from data. In turn, the latent variable T* is linked to the vector of covariates **W** as follows:

$$T_{i}^{*}=\beta \cdot W_{i}+\varepsilon$$

where **β** is again a set of N coefficients and ɛ is a random disturbance term that is supposed to follow a logistic distribution. Other choices of ɛ's parametric form lead to different types of models, e.g., Probit models in case of Gaussian noise.

The OL regression requires the estimation of N+K-1 parameters vs. (K-1) · N for the ML regression. Thus, OL regression requires fewer samples to fit than ML regression and for the same sample size it exhibits less variance in the estimation of the parameters. This is possible because OL regression exploits the outcome categories’ intrinsic order. On the other hand, OL is less general than ML since it can only be applied in case the outcome is ordinal.

### MC-CIT Experimental evaluation

An extensive evaluation over seven high-dimensional datasets was performed in order to assess MC-CIT performances. All datasets contain gene expression data that were produced in cancer-related studies and are available in the Gene Expression Omnibus website [13, 14].

Table 1 reports the datasets’ characteristics. Appendix A reports the preprocessing steps applied on the data.

**Table 1:** Characteristics of the datasets employed during the experimentation. For each dataset the following information are reported: Gene Expression Omnibus (GEO) ID, disease investigated during the study, type (categorical or ordinal) of outcome, number of classes, samples and variables.

GEO ID	Disease	Outcome	#Classes	#Samples	#Variables
GDS1329	Breast Cancer	Categorical	3	49	22215
GDS1962	Glioma	Ordinal	4	180	54613
GDS2373	Squamous Cell cancer	Ordinal	3	130	22284
GDS2547	Prostate cancer	Categorical	4	164	12646
GDS2855	Muscle diseases	Categorical	3	71	22645
GDS3233	Cervical cancer	Categorical	3	61	22283
GDS3257	Adeno carcinoma	Ordinal	3	101	22288

We contrasted the proposed MC-CIT against the prototypical Fisher Z and G^2^ test. All tests were embedded, in turn, within the local causal discovery, constraint-based MMPC algorithm [2].

MMPC identifies all the variables that cannot be made independent by the outcome, regardless by what conditioning set is considered. More formally, given a set of variables **D** and a target variable Y the MMPC algorithm applies an efficient heuristic in order to retrieve the set of variables **X** ⊆ **D** such that, for each X ∈ **X**, *Ind*(X, Y∣**Z**) does not hold for any conditioning set **Z** ⊆ **D**/{X}. Notably, under the assumption that the distribution of the data can be faithfully represented by a Bayesian Network [15] and that there are no statistical errors in the results of the independence tests, the variables identified by the MMPC algorithm correspond to the set of network nodes that are the parents and the children of the target variable (hence the name of the algorithm)^1^. Signatures retrieved by the MMPC algorithms proved to be particularly well-performing in term of predictive capabilities [4], even though the parents-children set consists of only a subset of the Markov Blanket.

The MMPC algorithm requires two different user-specified parameters: conditioning sets’ maximal size *k-max* and the significance threshold α. The latter is used for rejecting/accepting the null hypotheses of conditional independence. In our experimentation we vary α in {0.01, 0.05}, since these values are conventionally employed for assessing statistical tests’ significance. The *k-max* parameter indicates the maximal size allowed for **Z** in Test(X,Y∣**Z**), and it has an interesting graphical interpretation. Assuming that the distribution of the data can be faithfully represented by a Bayesian Network (BN), k-max represents our prior belief on the minimal number of outcome's neighbors necessary for d-separate the outcome from any other node (see [2] for further explanations). Notably, in our analyses we used only gene expression data, and gene regulatory networks are believed to be quite sparse [16]; consequently, during the experimentation we vary *k-max* in {3, 4}.

A simple data discretization procedure was applied in all analyses involving the G^2^ test: continuous values were assigned to one category among “low”, “medium” and “high”, depending by whether the values were falling respectively below, within or above the mean +/- the standard deviation of the respective variable.

We furthermore included the Lasso feature selection method [17] in our experimentation, in order to characterize MC-CIT performances against one of the currently cutting-edge feature selection algorithm. The Lasso algorithm belongs to the GLM class, and its objective function trades off the squared error on the training data with the sum of absolute values of the coefficients (norm-1 penalty). A parameter λ dictates the trade-off: a larger λ penalizes more for larger absolute weights. When λ is zero the standard least-squares fitting is obtained. As λ grows more and more variables obtain zero coefficients and are effectively removed from the model (are not selected). In this sense, the Lasso algorithm performs what is called embedded variable selection. Thus, larger λ values provide parsimonious models that may underfit, while low λ values provide more complex models that may overfit.

The optimal number of covariates that should be included into the model is not known in advance, and thus one should evaluate several lambda values sampled from a sufficiently wide interval. Some initial investigations pointed out that a lambda value of 0.2 was able to induce, on average over all the datasets included in this study, small models with approximately 5 (5.43) variables. A lambda value of 0.05 was instead leading to much larger models with ∼60 (61.86) variables. Thus, we decided to vary the penalty term λ in {0.05, 0.1, 0.15, 0.20}.

Notably, the Lasso algorithm can act as a feature selection procedure, as a regression algorithm, or as both at the same time. When the Lasso algorithm is used to select variables we refer to it as the *Lasso selection*, while *Lasso regression* will indicate the Lasso procedure employed as regression algorithm.

All feature selection methods were combined with three different classifiers, in order to produce testable predictions: Support Vector Machines (SVM, [18, 19]), Lasso regression and Multinomial Logistic regression (the latter was substituted by the Ordinal Logit regression in case of ordinal outcome). SVM were employed following the paradigm “one-vs.-all”, i.e., a different binary SVM model is fitted for each category of the outcome, while all other categories are considered as a unique alternative class. We employed the linear, polynomial (degree 2 and 3) and Gaussian kernel functions, and the user-specified cost parameter C was set to {1,10}. ML and OL regression do not require the user to specify any parameter, while Lasso regression penalty term λ was varied as reported above.

Summarizing, we employed four different feature selection strategies: the Lasso selection method and the MMPC algorithm coupled in turn with the MC-CIT, Fisher Z and G^2^ test. Each feature selection method was coupled in turn with three different classifiers: SVM, Lasso regression and ML/OL models.^2^

The nested-cross validation procedure [20] was employed for simultaneously (a) optimizing the parameters of both feature selection methods and classifiers (b) selecting the best classifier for each combination of dataset and feature selection method, and (c) providing unbiased estimates of the classification performances. Standard cross-validation consists in splitting the data in several non-overlapping folds; each fold is hold out in turn for testing purposes, while the rest of the data is used for training. Nested cross-validation is a generalization of the cross-validation procedure: an outer loop of cross-validation is employed for performance estimation, while an inner cross-validation loop is performed in each training-set for optimizing algorithms’ parameters. Accuracy (i.e., percentage of correct predictions) was employed as performance metric; the two-side binomial test [21] was employed for detecting statistically significant differences among methods’ accuracies. This statistical test is conceptually similar to the Pearson's Chi Squared test for 2x2 contingency tables; however, contrarily to the latter, the binomial test is an exact test.

## Results

Table 2 reports the main results of the experimentation. The best nested cross-validated performance is reported for each combination of dataset and feature selection method (“best” over the three classifiers). The first three columns show the results obtained by coupling the MMPC algorithm with the three different conditional independence tests. The next column reports the results obtained by the Lasso algorithm employed as feature selection method. The last column shows the results of the trivial classifier, i.e., the performances that one obtains by predicting the class that appears more frequently in the training set. Performances are reported as mean accuracy (averaged over nested-cross-validation outer-loop folders) ± standard deviation. Binomial test p-values for statistically comparing the difference in accuracy between MC-CIT and the other methods are reported in parentheses. Notice that the binomial test was always applied after pooling together all the predictions from the outer-loop of the nested-cross validation procedure, in order to increase the power of the test. The final row shows the “global” performances calculated by pooling together the predictions over all datasets. No method proved to outperform the trivial classifier for the GDS2373 dataset; we thus dropped the results related to this dataset.

**Table 2:** Nested cross validated results. Performances are reported as mean accuracy (averaged over nested-cross-validation outer loop) ± standard deviation. Values in parentheses are Binomial test p-values for comparing the respective accuracies against the corresponding MC-CIT performance. Columns “MC-CIT”, “Fisher Z test”, “G2 test” report the best accuracies obtained by the MMPC algorithm coupled with the respective conditional independence test. Column “Lasso” reports the best accuracies obtained by the Lasso feature selection method, while the last column report the baseline accuracy obtained by predicting the most frequent class (“Trivial classifier”). All methods performed similarly to the Trivial classifier for dataset GDS2373, thus its results are not shown. The last row reports the accuracies obtained by pooling together the predictions over all datasets.

Dataset	MC-CIT	Fisher Z test	G^2^ test	Lasso	Trivial cl.
GDS1329	0.959 ± 0.057	0.958 ± 0.065 (1.000)	0.958 ± 0.065 (1.000)	1.000 ± 0.000 (0.500)	0.551 ± 0.047 (0.000)
GDS1962	0.651 ± 0.061	0.650 ± 0.102 (1.000)	0.648 ± 0.092 (1.000)	0.699 ± 0.079 (0.108)	0.451 ± 0.024 (0.000)
GDS2547	0.656 ± 0.092	0.634 ± 0.116 (0.678)	0.712 ± 0.147 (0.389)	0.724 ± 0.084 (0.228)	0.391 ± 0.023 (0.000)
GDS2855	0.861 ± 0.112	0.652 ± 0.222 (0.003)	0.766 ± 0.159 (0.189)	0.861 ± 0.090 (1.000)	0.408 ± 0.035 (0.000)
GDS3233	0.984 ± 0.048	0.890 ± 0.114 (0.031)	0.981 ± 0.056 (1.000)	0.968 ± 0.065 (1.000)	0.460 ± 0.038 (0.000)
GDS3257	0.644 ± 0.096	0.607 ± 0.147 (0.523)	0.657 ± 0.152 (1.000)	0.642 ± 0.100 (1.000)	0.446 ± 0.042 (0.005)
**Global**	**0.732**	**0.685 (0.016)**	**0.735 (0.934)**	**0.765 (0.089)**	**0.438 (0.000)**

The number of selected feature is reported in Table 3. Averages and standard deviations are calculated on the outer-loop of the nested cross-validation procedure. Two-tailed t-test p-values for comparing MC-CIT performances against other methods results are reported in parentheses. The last row reports the average results over all datasets.

**Table 3:** Number of features selected in the outer-loop of the nested-cross validation procedure. Results are reported as mean ± standard deviation. Values in parentheses are p-values for statistically comparing MC-CIT against the other methods (two-tailed t-test). Column names follow the same schema of Table 2. The last row reports the average performances calculated over all datasets.

Dataset	MC-CIT	Fisher Z test	G^2^ Test	Lasso
GDS1329	2.000 ± 0.000	3.167 ± 0.408 (0.000)	35.833 ± 16.940 (0.001)	15.833 ± 4.446 (0.000)
GDS1962	6.100 ± 0.568	6.600 ± 0.699 (0.096)	8.200 ± 1.476 (0.001)	40.200 ± 35.888 (0.008)
GDS2547	14.200 ± 2.741	7.800 ± 1.033 (0.000)	6.100 ± 0.568 (0.000)	53.000 ± 27.897 (0.000)
GDS2855	3.700 ± 0.949	7.300 ± 1.252 (0.000)	28.500 ± 3.375 (0.000)	30.400 ± 10.233 (0.000)
GDS3233	2.000 ± 0.000	5.778 ± 1.202 (0.000)	34.222 ± 5.518 (0.000)	18.000 ± 5.196 (0.000)
GDS3257	4.900 ± 0.876	4.200 ± 1.549 (0.229)	4.700 ± 1.160 (0.669)	26.200 ± 20.531 (0.004)
**Global**	**5.483 ± 4.564**	**5.807 ± 1.810 (0.875)**	**19.593 ± 14.770 (0.049)**	**30.606 ± 14.072 (0.002)**

Some considerations now follow. First, MC-CIT always outperforms or equals the Fisher Z test. Most relevantly, the difference between the two methods is statistically significant for two datasets (GDS2855 and GDS3233) and when all predictions are pooled together (p-value < 0.05). Moreover, the MC-CIT method leads to the selection of fewer variables than the Fisher Z test for four datasets out of six, as well as on average over all datasets. The G^2^ test and the Lasso method usually achieve better results than MC-CIT in terms of performance (although not statistically significantly better at the level of 0.05), although in the expense of selecting more variables (statistically significantly). Specifically, on average the G^2^ test and the Lasso select about 4 and 6 times more variables, respectively.

All feature selection methods statistically significantly outperformed the trivial classifier. Moreover, all methods show small standard deviation values in terms of accuracy, denoting an appreciable stability. However, the Lasso and G^2^ test show a large variability in terms of number of selected variables.

Furthermore, we investigated whether the average number of selected features is somehow linked to datasets’ characteristics. For both Lasso selection and MMPC coupled with MC-CIT we found that the average number of selected variables is highly correlated with both sample size and number of outcome classes (Pearson correlation ρ > 0.75). A similar effect is found also for the Fisher Z test, though not equally strong (ρ ≈ 0.55). MMPC coupled with the G^2^ test tends to select fewer variables when the sample size and the number of outcome classes increase (ρ < -0.65). The MMPC algorithm does not include in the signature any variable whose (conditional) association with the outcome cannot be assessed. As the number of outcome's classes increases, the G^2^ test has less power, and our implementation of the G^2^ test forgoes assessing conditional independencies when the expected power is excessively low (see [22] for further details on the heuristic employed for ensuring a sufficient power for the G^2^ test). Finally, the number of selected features does not seem to be correlated with the total number of available features (∣ρ∣ < 0.25 in all cases).

No feature selection method showed to constantly produce better results when coupled with a particular classifier. However, the Lasso regression proved to be the classifier that most often provides the best performances, followed by the SVM and the multinomial logistic models.

## Discussion

In this work we introduced a new conditional independence test, namely the Multinomial-Logistic Conditional Independence Test (MC-CIT), explicitly devised for being coupled with constraint-based methods for biomarker signature identification in multi-class outcome data. We performed an extensive evaluation of the new test on seven different gene-expression datasets, contrasting the MC-CIT against the Fisher Z test, which is, to the best of our knowledge, the most commonly-employed CIT for multi-class problems with continuous regressors. We further contrasted the new test against a prototypical CIT for discrete data (after performing an appropriate discretization of the continuous regressors) and against the provably well performing Lasso algorithm.

The results confirmed our initial hypothesis: *for multi-class outcome problems MC-CIT allows the identification of smaller and better performing* (in a statistically significant way) *signatures, with respect to the widely employed Fisher Z test*. This finding suggests employing the MC-CIT instead of the Fisher Z test for feature selection tasks with multi-class outcome.

The GDS2547 datasets is the only case when the MC-CIT selects a significantly larger set of features with respect to the Fisher Z test. Interestingly, a visual inspection of the GDS2547 data revealed the presence of strong linear relationships between the outcome and the features selected by MMPC coupled with the Fisher Z test. The latter assumes that (and has more statistical power when) the data are (approximately) linear. Thus the presence of linear relationships in the data can be a plausible cause for Fisher Z test better performances (in terms of number of selected features).

Somewhat surprising, the G^2^ test proved to slightly, non-significantly outperform the MC-CIT in few cases, at the cost of selecting four times more variables (on average). This behavior has not a clear explanation yet.

No feature selection method was able to identify a predictive signature for the GDS2373 dataset. A possible explanation is that the transcriptomic data reported in this dataset are not informative with respect to the outcome (tumor stage). Interestingly, the researchers that first introduced this datasets reached similar conclusion. In their analysis, the data were clustered according to an unsupervised hierarchical clustering method, and the resulting clustering groups were not associated with the disease stage [23].

Finally, MMPC coupled with MC-CIT achieves levels of performance that are fairly competitive with the Lasso selection results. The Lasso algorithm provides moderately more accurate models that are, at the same time, more complex than the ones provided by the MMPC coupled with MC-CIT. However, these results may depend by the parameter configurations tested in our experimentations: testing different λ, α or *k-max* values may lead to different results.

One limitation of this study is that it is not possible to compare Fisher Z test and MC-CIT computational requirements, since we did not make any attempt to optimize the implementation of the new test. Furthermore, our experimentations were limited to Gene Expression data only. However, we expect that the main conclusions of this study should hold for any other type of “omics” data (e.g., RNA-seq, miRNA, methylation data) that share the main characteristics of the datasets involved in our experimentations: continuous measurements, high dimensionality and multi-class outcome.

In conclusion, our findings indicate that MC-CIT should be preferred to the Fisher Z test in studies aiming at identifying biomarker signatures in “omics” data with multi-class outcome. Furthermore, MC-CIT is a valid alternative against the G^2^ test, which requires an additional preprocessing step of data discretization. Finally, when compared against the Lasso selection MMPC coupled with MC-CIT demonstrated to be able to select smaller and statistically equally predictive biomarker signatures, within the scope of our experiments.

## Footnotes

1 More precisely, we employed the MMPC version “without symmetry correction”, that can return a superset of the parent and children set (see [2] for further details).

2 We also employed the Lasso algorithm for directly producing classification models without a preliminary selection of the variables. The results of this approach were statistically indistinguishable from the results obtained by using the Lasso algorithm as a feature selection procedure; consequently, we do not report them.

## Appendix A

Measurements were log2 transformed in all datasets. In each training set, missing values were substituted with the mean value of their respective variables, and each variable was scaled in order to have a zeroed mean and unitary standard deviation. Test sets underwent the same preprocessing steps, using the mean and standard deviation values previously calculated on the respective training sets. Some datasets underwent additional preprocessing steps:

GDS2855: only outcome classes with 20 or more subjects (namely “normal”, “juvenile dermatomyosis” and “Emery-Dreifuss muscular dystrophy”) were retained for subsequent analyses.

GDS3257: only three subjects belonged to the outcome class “Stage IV” and were excluded from the analyses.
